# Difference of concentration of placental soluble fms-like tyrosine kinase-1(sFlt-1), placental growth factor (PlGF), and sFlt-1/PlGF ratio in severe preeclampsia and normal pregnancy

**DOI:** 10.1186/s13104-015-1506-0

**Published:** 2015-10-04

**Authors:** Jeffry Iman Gurnadi, Johannes Mose, Budi Handono, Mieke H. Satari, Anita Deborah Anwar, Prima Nanda Fauziah, A. Yogi Pramatirta, Dwi Davidson Rihibiha

**Affiliations:** Department of Obstetrics and Gynecology, Faculty of Medicine, Padjadjaran University, Bandung, Indonesia; Department of Microbiology, Faculty of Dentistry, Padjadjaran University, Bandung, Indonesia; Department of Biology, School of Life Sciences and Technology, Bandung Institute of Technology, Bandung, Indonesia; Department of Biotechnology, School of Life Sciences and Technology, Bandung Institute of Technology, Bandung, Indonesia

**Keywords:** Normal pregnancy, Severe preeclampsia, PlGF, sFlt-1

## Abstract

**Background:**

Placental soluble fms-like tyrosine kinase-1 (sFlt-1) which is an antagonist of vascular endothelial growth factor and placental growth factor (PIGF), is considered as one of etiology factors cause endothelial damage in preeclampsia due to increase of sFlt-1 level that change vascular endothelial integrity. This study aims to analyze the difference of sFlt-1 and PlGF concentration in severe preeclampsia and normal pregnancy, and the correlation between both in occurrence of severe preeclampsia.

**Method:**

This is case control study involving 18 subjects with severe preeclampsia and 19 subjects with normal pregnancy as controls who met inclusion and exclusion criteria. Concentration of sFlt-1 and PlGF are measured with ELISA. Statistical analysis is performed with Chi square test, Fisher’s exact test, T test, Mann–Whitney test, and Spearman’s rank correlation test.

**Results:**

This study results in no significant difference in characteristics of gestational age, and parity in both study groups. Median concentration of sFlt-1 in severe preeclampsia is higher (20,524.75 pg/mL) compared with normal pregnancy (6820.4 pg/mL). Concentration of PlGF is lower in severe preeclampsia (47 pg/mL) compared with normal pregnancy (337 pg/mL). sFlt-1 concentration is higher in severe preeclampsia compared to normal pregnancy. PlGF concentration is lower in severe preeclampsia compared to normal pregnancy. Ratio of sFlt-1 and PlGF concentration is significantly correlated in both severe preeclampsia and normal pregnancy.

**Conclusions:**

There is a significant negative correlation between the concentration of sFLt-1 and PlGF in normal pregnancy.

## Background

Gestational hypertension is one of some most common complications in pregnancy, labor, or postpartum which increases maternal and perinatal morbidity and mortality. Severe preeclampsia is a pregnancy-specific syndrome characterized by systolic blood pressure of ≥160 mmHg and diastolic blood pressure of ≥110 mmHg, with qualitative proteinuria of > +1 or 0.3 g/24 h. [1, 2] World Health Organization (WHO) in 2006, reported that almost 16 % of 3201 mortality caused by pregnancy was contributed by gestational hypertension [3]. According to data from National Center for Health Statistics in USA, gestational hypertension occurs in 150,000 women or 3.7 % of all pregnancy [1]. National Vital Statistics recorded that incidence of preeclampsia increases by 40 %. Survey done by SKDI in 2007 found that preeclampsia/eclampsia contributes to about 24 % maternal mortality in Indonesia, makes it the second cause of maternal death in Indonesia. In Dr. Hasan Sadikin Hospital, Bandung, preeclampsia was reported in 3.5 % cases and eclampsia in 2.8 % cases in 2006, while in 2008–2010 preeclampsia was reported in 4.0–10.4 % and eclampsia in 2.3–4.3 % [4–10].

It has been hypothesized that the underlying mechanism of preeclampsia involve genetic factor, immunologic factor, vascular disease, and conditions in which excessive trophoblast were unable to invade spiral artery in the early phase of the first trimester. These cause spiral artery to dilate inadequately and disturbance in endothelial and muscular wall of blood vessel, leaves the blood vessel be smaller in diameter, leading to diminished perfusion and placental ischemia [11–13]. Placental ischemia causes elevated concentration of placental soluble FMS-like tyrosine kinase-1 (sFlt-1), which is an antiangiogenic factor produced by hypoxic placental cells [14–17]. sFlt-1 is believed as an etiologic factor of endothelial damage in preeclampsia. Elevated concentration of sFlt-1 will modify endothelial integrity of blood vessel, causing blood–brain-barrier damage which lead to hepatic and brain edema, as well as hypertension and proteinuria encountered in preeclamptic patients. sFlt-1 can also be found in normal pregnancy after the 35th week [22]. sFlt-1 found in preeclamptic white people was about 4383 ± 657 pg/ml, while being 1643 ± 940 pg/ml in normal pregnancy [18–20] Elevation of sFlt-1 concentration was found weeks before clinical onset of hypertension and proteinuria [21–23].

Placental growth factor (PlGF), a type of vascular endothelial growth factor (VEGF), is a dimeric glycoprotein amino-acid residue which is an important local mediator in angiogenesis. PlGF is produced by placenta (especially by cytotrophoblast, syncytiotrophoblast, and extra-filamentous trophoblast), endothel of human umbilical vein, and choriocarcinoma cells. Alteration of its circulating concentration may affect the balance of placental vessel angiogenesis, thus its presence is often associated with preeclampsia occurrence because it reflects abnormality in placental development [22, 24, 25]. Studies have believed that PlGF alteration starting from 10 to 11th gestational week is a predisposing factor to preeclampsia in advanced pregnancy [23–25]. sFlt-1 is antagonistic to PlGF. Alteration of sFlt-1 and PlGF would cause imbalance of angiogenesis, followed by failure in trophoblastic invasion and physiological remodeling of spiral artery, thus placental hypoxia ensue [22, 23].

These raise a question whether disturbance in the balance of sFlt-1 and PlGF concentration was correlated with severity of clinical manifestation in patients. This study aims in comparing the concentration of sFlt-1 and PlGF in preeclamptic woman and normal pregnancy in the third trimester, and its correlation with patients’ clinical manifestations. The result is expected to be able in correlating clinical manifestation with theory of preeclampsia etiology, and to be used as a marker in detecting preeclampsia.

## Results and discussion

Characteristic data, systolic and diastolic blood pressure, measurement of proteinuria, and blood to be measured for PlGF and sFlt-1 with ELISA method in Prodia laboratory Bandung were collected from both study groups. Characteristics of parity, and gestational age were compared for homogeneity.

As shown in Table 1, there is no significant difference in maternal parity (p = 0.105, p > 0.05) and gestational age (p = 0.221, p > 0.05) between the two groups. Characteristics of subjects are therefore homogenous. It has been documented that younger age, primigravida, and gestational age bring risk of preeclampsia as confounding factor [1–3]. Incidence of preeclampsia is higher in young age and nulipara, while it also increases with advanced maternal age\due to increased risk of chronic hypertension, particularly in age older than 35 years old [3]. Characteristics of subjects in this study are homogenous in maternal age, gestational age, and parity.

**Table 1:** Characteristic of study subjects by parity and gestational age

Characteristics	Group		p value
	Preeclampsia (n = 18)	Normal (n = 19)	
Parity			
Primi	18	15	0.105*
Multi	0	4	
Gestational age (weeks)			
37	8	10	0.221
38	6	2	
39	4	7	
Mean (±SD)	37.8 (0.8)	32.4 (5.4)	
Median	38	37	
Range	37–39	37–39	

p value is analyzed with Chi square test, except for the maternal age (analyzed with t test) and parity (analyzed with Fisher’s exact test)*

Referring to the theory of preeclampsia and its pathophysiology, which is imbalance of pro- and antiangiogenic factor, correlation between sFlt-1 and PlGF concentration is important to be noted. There is a negative correlation between them: the higher is the sFlt-1, the lower is the PlGF [3].

Table 2 shows that concentration of sFlt-1 is significantly higher in the group with severe preeclampsia (p < 0.001), while PlGF in that group is significantly lower than control (p < 0.001). Table 2 shows a rational correlation between elevated concentration of sFlt-1 and reduced concentration of PlGF in the group with severe preeclampsia. Ratio of sFlt-1 and PlGF concentration is significantly correlated in both severe preeclampsia and normal pregnancy (p < 0.001). The higher the sFlt-1, the lower is the PlGF, with the difference of severe preeclampsia group of almost 15 times greater than the normal pregnancy group. Referring to the theory of preeclampsia pathogenesis, hypoxic placenta will release sFlt-1, which is a free soluble, into maternal circulation and this will be followed by reduction of free PlGF. This change can be noticed in preeclamptic patient weeks before its onset [3].

**Table 2:** Comparison of sFlt-1 and PlGF concentration in severe preeclampsia and normal pregnancy

Characteristics	Group		p value
	Preeclampsia (n = 18)	Normal (n = 19)	
sFlt-1 (pg/dl)			<0.001
Median	20,524.75	6820.4	
Range	1430.5–28,540.5	1657.6–18,859.6	
PlGF (pg/dl)			
Median	47	337	<0.001
Range	10–1361	54–1156	

p value is analyzed with Mann–Whitney test; sFlt-1 is analyzed with t test

It has been hypothesized that sFlt-1 concentration would continue to elevate as placental hypoxia advances, and this is associated with clinical manifestation of preeclampsia, which are elevated blood pressure and proteinuria [26]. In the group with severe preeclampsia, PlGF concentration continues to reduce, reflecting a negative correlation with sFlt-1. Elevation of circulating sFlt-1 concentration would reduce PlGF concentration until endothelial dysfunction ensues. This condition can be noted a few weeks before preeclampsia, therefore can be used as a diagnostic tool of preeclampsia [3, 18, 19, 27]. When elevated sFlt-1 is found in a woman without clinical manifestation of preeclampsia, the woman will have preeclampsia in the next few weeks, theoretically [19]. Parenteral administration of sFlt-1 on animals had been documented to elevate blood pressure, proteinuria, and glomerular endotheliosis, which are similar with clinical manifestations of preeclampsia. Preventing damage in glomerular endothelium can be achieved by reduction VEGF (VEGF-inhibitor administration) [3, 20].

Table 3 shows there is a negative correlation between elevated concentration of sFlt-1 and PlGF concentration. It is not significant in severe preeclampsia (r = −0.347), but very significant in normal pregnancy (r = −0.508). With both are combined, there is a very significant correlation in sFlt-1/PlGF ratio (r = 0.186). There is correlation between change of sFlt-1/PlGF concentration in severe preeclampsia and normal pregnancy, but it is not significant (0 < r < 1). This proves that elevated sFlt-1 concentration as antiangiogenic factor and reduction of PlGF as proangiogenic factor occur in pathogenesis of preeclampsia [28, 29, 31]. sFlt-1 concentration in preeclamptic women was found to be elevated, this leads to antagonistic process to VEGF and PlGF. As mentioned above, PlGF is produced by placenta and its concentration elevates during pregnancy without preeclampsia [30].

**Table 3:** Correlation of sFlt-1/PlGF ratio in severe preeclampsia and normal pregnancy

Characteristic	Group		r value
	Preeclampsia (n = 18)	Normal (n = 19)	
sFlt-1/PlGF (pg/ml)			
Median	303.0	20.2	0.186
Range	4.71–2646.57	2.49–238.73	
r value	−0.347	−0.508	

Analyzed with Mann–Whitney test

Not significant correlation of sFlt-1/PlGF might be caused that etiology of preeclampsia is multifactorial. A theory has been explained in a study by Aggarwal et al. that preeclampsia is not only caused by sFlt-1 elevation and PlGF reduction, but also by endothelin-1 (ET-1), a potent peptide with vasoconstrictive effect, which is released by endothelium and muscles of blood vessel [3, 17].

Further studies including variation in parity, gestational age, maternal age, and clinical manifestations to learn sFlt-1 and PlGF concentration according to parity, gestational age, and maternal age, and studies with larger number of samples, are encouraged.

## Study limitation

In this study, samples used were small and it might not reflect the real findings.

## Conclusion

Concentration of sFlt-1 in severe preeclampsia is higher than its concentration in normal pregnancy, while PlGF concentration in severe preeclampsia is lower than its concentration on normal pregnancy. Ratio of sFlt-1/PlGF is positively-correlated in normal pregnancy.

Concentration of sFlt-1 is negatively-correlated with normal pregnancy. Concentration of sFlt-1 is negatively-correlated with proteinuria degree in severe preeclampsia. Ratio of sFlt-1/PlGF is positively-correlated with diastolic blood pressure and proteinuria in severe preeclampsia.

## Methods

This is a case control study. Pregnant women with singleton living-intrauterine fetus with gestational age of ≥37 weeks according to the last day of menstrual period and medical record presented to Dr. Hasan Sadikin hospital, Cibabat hospital, and Astana Anyar hospital in January 2011 to June 2011 were included in this study. Those with normal blood pressure and negative proteinuria were included in control group (normal pregnancy), while those who meet criteria of preeclampsia with positive proteinuria were included in the preeclampsia group. Fetal congenital malformations confirmed with USG, mother having infection or history of chronic diseases (renal, cardiac, hypertension, diabetes mellitus, hypothyroidism, peptic ulcer, etc.), who smokes or drink alcohol, and pregnancy with fetal growth restriction, were excluded. Subjects were taken consecutively, consisted of 18 preeclamptic patients and 19 women with normal pregnancy as control group.

Patients with preeclampsia (18 samples) and normal pregnancies as controls (19 samples) were managed according to guidelines therapy of Obstetric and Gynecology Faculty of Medicine Padjadjaran University Hasan Sadikin Hospital (FKUP/RSHS), Bandung, through some tests includes: (1) anamnesis; name, age, address, parity, first day of last haid, gestational age, previous hypertension history, and current pregnancy disease. (2) blood pressure was measured by using sphigmomanometer to patients who were treated lying on its left side for 15 min. (3) 6 ml blood sample was taken from peripheral blood before birth, centrifuge at 1600*g* 10′ 4 °C. Blood sample was withdrawn and then kept in −20 °C temperature. (4) Levels of sFlt-1 and PlGF were performed by *high sensitivity indirect sandwich enzyme*-*linked immunosorbent assay* (ELISA) in Prodia laboratory in Jakarta afterwards.

Data analyzing was begun by performing normality test. Categorical data were analyzed with Chi square or Fisher’s exact test when the expected value was less than 5. Normally-distributed data were compared with T test, while not-normally-distributed ones were analyzed with Mann–Whitney test. Ratio of proteins was analyzed with Spearman’s rank correlation test. Data analysis was performed with SPSS for Windows version 15.0, with 85 % confidence interval and p value of <0.05.
